# Clinical Effectiveness, Safety, and Implementation of Artificial Intelligence-Assisted Insulin Management Systems in Type 1 and Type 2 Diabetes: A Systematic Review of Randomized Controlled Trials

**DOI:** 10.3390/diseases14100378

**Published:** 2026-10-13

**Authors:** Victória Dogani Rodrigues, Virgínia Maria Cavallari Strozze Catharin, Mauro Audi, Eliana de Souza Bastos Mazuqueli Pereira, Jesselina Francisco dos Santos Haber, Débora Audi, Vitor Cavallari Strozze Catharin, Maria Angélica Miglino, Lucas Fornari Laurindo

**Affiliations:** 1 Department of Biochemistry and Pharmacology, School of Medicine, Faculdade de Medicina de Marília (FAMEMA), Marília 17519-030, SP, Brazil; 2 Graduate Program in Structural and Functional Interactions in Rehabilitation, School of Medicine, Universidade de Marília (UNIMAR), Marília 17525-902, SP, Brazil; 3 Graduate Program in Medical Sciences, Faculdade de Medicina, Universidade de São Paulo (USP), São Paulo 01246-903, SP, Brazil

**Keywords:** artificial intelligence, diabetes mellitus, insulin therapy, insulin titration, automated insulin delivery, closed-loop systems, machine learning, randomized controlled trials, systematic review

## Abstract

Background: Artificial intelligence (AI) is increasingly being integrated into insulin management through decision-support systems, adaptive bolus calculators, conversational platforms, and automated insulin delivery (AID) technologies. However, the clinical effectiveness and safety of AI-assisted insulin management remain uncertain. Objective: To systematically evaluate evidence from randomized controlled trials (RCTs) investigating AI-assisted insulin management systems in individuals with type 1 or type 2 diabetes. Methods: A systematic literature review was conducted following PRISMA guidelines. PubMed/MEDLINE, Embase, and Cochrane CENTRAL were searched for RCTs evaluating AI-enabled insulin management interventions in insulin-treated individuals with diabetes. Eligible studies compared AI-based systems with standard care, clinician-guided insulin titration, conventional bolus calculators, or other AID algorithms. Outcomes included glycemic control, insulin titration, safety, and implementation-related measures. Owing to substantial heterogeneity, findings were synthesized narratively. Results: We included seven RCTs published between 2020 and 2025. Interventions comprised clinical decision-support systems, adaptive bolus calculators, conversational AI platforms, neural-network-based artificial pancreas algorithms, and exercise-aware AID systems. AI-assisted interventions consistently demonstrated favorable safety profiles, with no increase in severe hypoglycemia, diabetic ketoacidosis, or serious adverse events. Some systems, particularly fully automated closed-loop platforms and conversational AI-guided insulin titration, showed improvements in time in range, glucose control, adherence, and insulin optimization. User acceptance and adherence emerged as important determinants of effectiveness. Conclusions: Current randomized evidence suggests that AI-assisted insulin management systems are safe, feasible, and capable of achieving glycemic outcomes comparable to conventional care. Although certain technologies demonstrate promising clinical benefits, the evidence base remains limited by small sample sizes, short follow-up durations, and heterogeneity across interventions. Larger, long-term randomized trials are needed to establish comparative effectiveness, optimize human–AI interaction, and support broader clinical implementation.

## 1. Introduction

Diabetes mellitus remains one of the most significant global public health challenges, affecting hundreds of millions of individuals worldwide and contributing substantially to morbidity, mortality, and healthcare expenditure. Despite advances in glucose monitoring technologies and insulin delivery systems, achieving and maintaining optimal glycemic control remains difficult for many people with diabetes [1,2]. Insulin therapy is highly effective but requires complex and dynamic dose adjustments that depend on numerous factors, including glucose trends, meal composition, physical activity, insulin sensitivity, and behavioral adherence [3,4,5]. Inadequate insulin titration can result in persistent hyperglycemia and increased risk of long-term microvascular and macrovascular complications, and episodes of hypoglycemia that adversely affect quality of life and treatment adherence [6,7].

Recent years have witnessed rapid growth in the application of artificial intelligence (AI) to diabetes management. AI encompasses a broad range of computational techniques, including machine learning, neural networks (NN), adaptive algorithms, predictive analytics, and intelligent decision-support systems capable of identifying patterns within large and complex datasets [8,9]. In the context of insulin management, AI technologies are increasingly being integrated into clinical decision support platforms, automated insulin delivery (AID) systems, adaptive bolus calculators, and patient-facing digital health tools [10,11]. By continuously processing glucose measurements, insulin dosing histories, physiological signals, and behavioral data, these systems aim to deliver personalized insulin recommendations, automate dose adjustments, and reduce the cognitive burden associated with diabetes self-management.

The potential advantages of AI-assisted insulin management are considerable [12]. Automated and adaptive systems may improve glycemic outcomes by responding more rapidly and consistently than conventional approaches, potentially reducing therapeutic inertia, minimizing clinician workload, and supporting individualized treatment strategies. Emerging technologies have also expanded beyond traditional glucose-responsive algorithms to incorporate contextual information such as physical activity, wearable sensor data, and patient interactions through conversational interfaces [13]. These developments have generated increasing interest in whether AI-enabled systems can achieve superior or at least comparable clinical outcomes to expert-guided care while maintaining safety.

However, the clinical effectiveness of AI-assisted insulin management remains uncertain. Existing technologies vary substantially in their underlying architecture, degree of automation, intended user population, and clinical implementation. Some systems function primarily as decision-support tools requiring clinician or patient oversight, whereas others operate as fully or partially automated closed-loop insulin delivery platforms. Furthermore, evidence from individual studies has been heterogeneous, with some trials reporting improvements in glycemic metrics such as time in range (TIR), whereas others have demonstrated only noninferiority or feasibility without significant clinical benefit. Concerns regarding algorithm transparency, user acceptance, adherence to AI-generated recommendations, regulatory oversight, and long-term safety also remain important considerations for widespread adoption.

Previous reviews have primarily examined AI applications across the diabetes continuum, including diagnosis, risk prediction, complication screening, and glucose monitoring. In contrast, the present review focuses specifically on randomized evidence evaluating AI systems directly involved in insulin management decisions. Although reviews have examined broader applications of AI in diabetes care, the evidence specifically derived from randomized controlled trials (RCTs) evaluating AI-assisted insulin management systems has not been comprehensively synthesized. Randomized trials provide a high level of evidence for assessing clinical efficacy and safety and are particularly important in evaluating technologies that directly influence insulin dosing decisions. Given the increasing diversity of AI-driven insulin management approaches and their growing integration into clinical practice, a focused evaluation of randomized evidence is warranted.

Therefore, the aim of this systematic review was to evaluate RCTs investigating AI-assisted insulin management systems in individuals with type 1 or type 2 diabetes. We sought to examine the effects of these interventions on glycemic control, insulin titration, safety outcomes, and implementation-related measures, while identifying common patterns, strengths, limitations, and future directions for the integration of AI into insulin therapy. In this review, AI-assisted insulin management was defined as the use of computational systems that incorporate AI techniques, including machine learning, NN, adaptive algorithms, case-based reasoning, or data-driven predictive models, to support insulin dosing decisions, glucose prediction, or AID. Systems based solely on fixed rule-based algorithms without adaptive or learning capabilities were not considered AI-assisted unless they incorporated an AI-based component as part of the decision-support or insulin delivery framework.

## 2. Materials and Methods

### 2.1. Study Design and Reporting Standards

This systematic review was conducted to synthesize evidence from RCTs evaluating AI-assisted insulin management systems in individuals with diabetes mellitus. The review followed established methodological guidance for systematic reviews of healthcare interventions and was structured in accordance with the Preferred Reporting Items for Systematic Reviews and Meta-Analyses (PRISMA) framework [14].

The aim of this systematic review was to evaluate the effectiveness of AI-assisted insulin management systems in individuals with diabetes mellitus, focusing on glycemic control, including TIR, time above range (TAR), time below range (TBR), mean glucose, glycemic variability, and insulin requirements. We also assessed safety outcomes, including hypoglycemia and adverse events, as well as implementation-related outcomes such as adherence, feasibility, and user acceptance.

This systematic review was not prospectively registered in PROSPERO. Nevertheless, the review was conducted according to a predefined protocol and followed the PRISMA reporting guidelines.

### 2.2. Eligibility Criteria

Studies were included if they met the following criteria:

(1) Study design: RCTs, including parallel-group and crossover designs.
(2) Population: Adults or adolescents with type 1 diabetes (T1D) or type 2 diabetes (T2D) receiving insulin therapy.
(3) Intervention: AI-enabled or algorithm-driven insulin management systems, including but not limited to clinical decision support systems, AID systems, neural-network-based controllers, adaptive bolus calculators, or conversational AI platforms for insulin titration.
(4) Comparator: Standard care, physician-guided insulin titration, conventional bolus calculators, or other AID algorithms.
(5) Outcomes: At least one glycemic outcome measured using continuous glucose monitoring (CGM) or laboratory markers, including TIR, TAR, TBR, glycated hemoglobin (HbA1c), mean glucose, glycemic variability, or safety outcomes such as hypoglycemia or adverse events.

Studies were excluded if they were non-randomized, observational, simulation-only without clinical evaluation, lacked a comparator arm, or did not report clinically relevant glycemic outcomes.

### 2.3. Information Sources and Search Strategy

A comprehensive literature search was conducted across major biomedical databases, including PubMed/MEDLINE, Embase, and the Cochrane Central Register of Controlled Trials (CENTRAL), to identify relevant RCTs evaluating AI-assisted insulin management systems in individuals with diabetes mellitus. The searches were conducted through 24 July 2026, and all databases were searched from inception to the search date. Additional records were identified through manual screening of the reference lists of included studies and relevant reviews. The search strategy combined controlled vocabulary (e.g., MeSH terms) and free-text terms related to “diabetes,” “insulin,” “artificial intelligence,” “machine learning,” “decision support systems,” “closed-loop insulin delivery,” and “randomized controlled trial.” No restrictions were applied based on publication year, although only studies published in English were included.

### 2.4. Study Selection

All identified records were imported into a reference management software (EndNote 2025), and duplicate records were removed prior to screening. Two reviewers independently screened the titles and abstracts of all retrieved records against the predefined eligibility criteria. Studies considered potentially eligible underwent independent full-text assessment by the same two reviewers. Any disagreements regarding study eligibility were resolved through discussion and consensus. The study selection process is summarized in a PRISMA flow diagram. The final included studies comprised randomized evaluations of AI-based insulin management systems conducted in inpatient and outpatient settings.

### 2.5. Data Extraction

Two reviewers independently extracted data using a standardized data extraction framework. Any discrepancies were resolved through discussion and consensus before the final dataset was established. Extracted variables included:

- Study characteristics (author, year, country, design, and duration);
- Participant characteristics (sample size, age, diabetes type, baseline glycemic control);
- Intervention details (type of AI system, architecture, and level of automation);
- Comparator description;
- Primary and secondary outcomes (CGM-derived metrics, insulin dose adjustments);
- Safety outcomes (hypoglycemia, severe hypoglycemia, hyperglycemia, adverse events);
- Key implementation outcomes (e.g., adherence, acceptance of recommendations, feasibility where reported).

Where multiple publications reported on the same intervention, the most complete dataset was used.

### 2.6. Risk of Bias Assessment

Risk of bias in individual studies was independently assessed by two reviewers using the Cochrane Risk of Bias 2 (RoB 2) tool for randomized trials [15]. The following domains were evaluated:

- Bias arising from the randomization process;
- Bias due to deviations from intended interventions;
- Bias due to missing outcome data;
- Bias in measurement of outcomes;
- Bias in selection of the reported result.

The standard RoB 2 tool was applied to all included trials, including crossover trials; therefore, period and carryover effects were not assessed using the dedicated crossover version.

### 2.7. Data Synthesis

Given the heterogeneity in study populations (T1D vs. T2D), intervention types (decision support systems, closed-loop AID, bolus calculators, and conversational AI), study designs (parallel vs. crossover), and outcome reporting, a quantitative meta-analysis was not performed. Instead, a narrative synthesis was conducted. Findings were grouped according to:

- Type of AI system (closed-loop insulin delivery, decision support/bolus calculators, conversational AI);
- Clinical setting (inpatient vs. outpatient vs. free-living conditions);
- Level of automation and user interaction;
- Glycemic efficacy outcomes (TIR, TBR, mean glucose);
- Safety and implementation outcomes (hypoglycemia, adverse events, user adherence, and system acceptance).

This approach allowed comparison of performance trends across heterogeneous AI modalities while preserving study-specific context.

Although quantitative synthesis was considered, meta-analysis was not performed because the included studies demonstrated substantial clinical and methodological heterogeneity in AI intervention type, patient populations, comparators, outcomes, and follow-up duration. Furthermore, the small number of eligible studies precluded reliable assessment of publication bias.

### 2.8. Outcomes of Interest

The outcomes of interest in this review included glycemic control, primarily assessed using TIR (70–180 mg/dL or equivalent), as well as other glycemic outcomes, including TBR, TAR, mean glucose, glycemic variability, and insulin dose requirements. Safety outcomes, including severe hypoglycemia and other adverse events, and implementation-related outcomes such as adherence to AI recommendations, feasibility, and user acceptance, were also assessed.

## 3. Results

**Figure 1:**
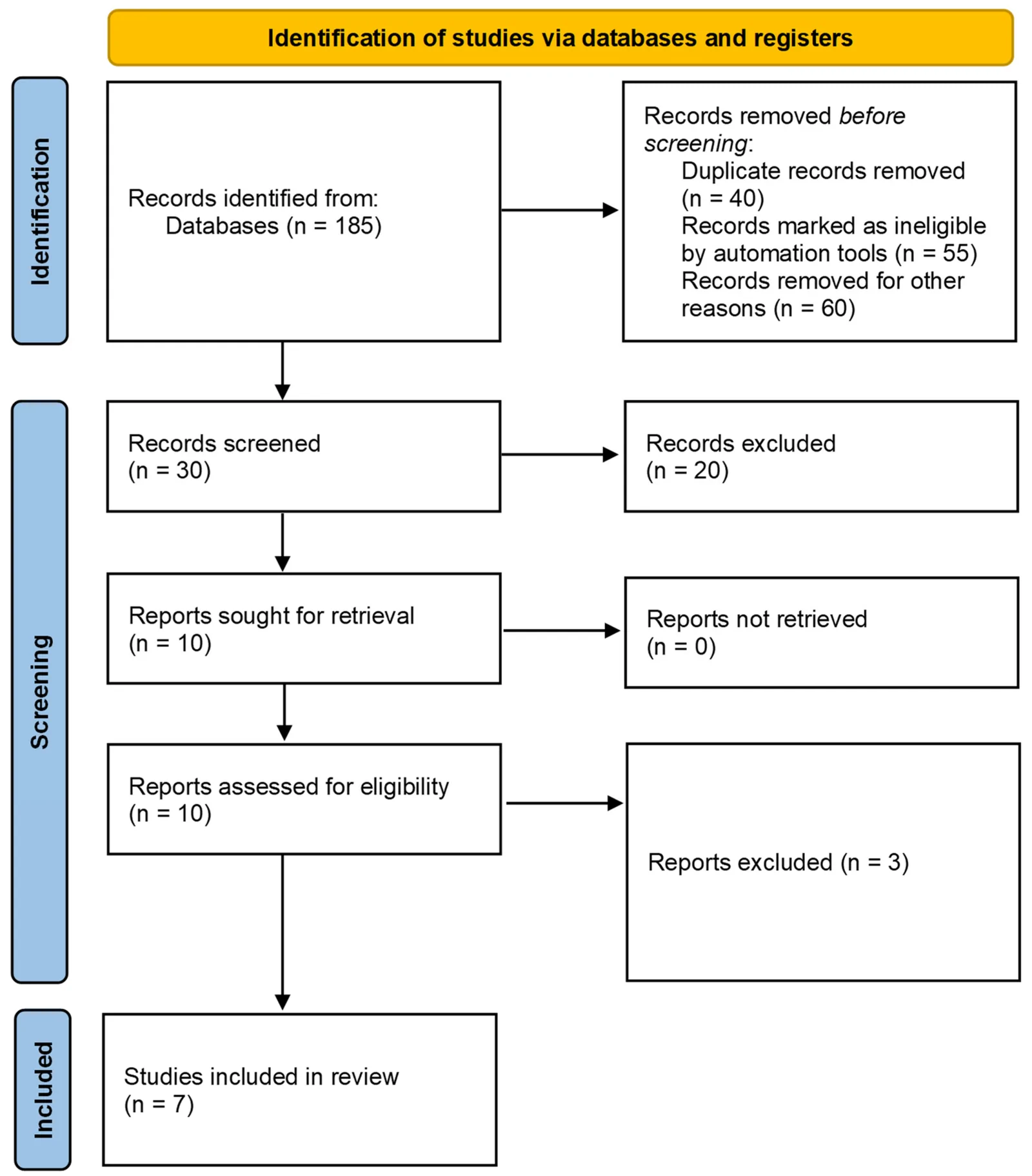
PRISMA flow diagram of study selection [14].

The study selection process followed the PRISMA guidelines [14]. As shown in Figure 1, studies were identified through systematic database searches and subsequently underwent screening, eligibility assessment, and inclusion according to the predefined criteria. After removal of duplicates and exclusion of records that did not meet the eligibility requirements, seven RCTs were included in the qualitative synthesis.

The included studies were published between 2020 and 2025 and evaluated AI-assisted insulin management interventions in adults with type 1 or type 2 diabetes. The interventions encompassed a diverse range of AI-based technologies, including clinical decision support systems, adaptive bolus calculators, conversational AI platforms, neural-network-based artificial pancreas algorithms, and exercise-aware AID systems.

The studies were conducted in China, the United States, the United Kingdom, and Spain, and included both hospitalized and ambulatory populations. Overall, the seven trials assessed outcomes related to glycemic control, insulin titration, treatment adherence, safety, feasibility, and user acceptance. Studies were excluded during full-text review if they were not RCTs, did not evaluate an AI-based intervention for insulin management, lacked relevant clinical outcomes, or represented non-original research such as reviews, protocols, editorials, or conference abstracts. Given that five of the seven included RCTs involved adults with T1D, we additionally examined this subgroup descriptively. However, quantitative pooling was not considered appropriate because of substantial heterogeneity in AI architecture, comparator interventions, study settings, and outcome reporting.

## 4. Discussion

Table 1 summarizes RCTs evaluating AI-assisted insulin management systems in patients with diabetes, highlighting heterogeneity in AI system design (e.g., decision support, closed-loop control, and adaptive bolus algorithms), clinical populations, comparator treatments, study durations, and primary glycemic and safety endpoints. The included interventions represent a spectrum of AI integration, ranging from adaptive decision-support systems to neural-network-based and AI-augmented AID approaches. Therefore, the term AI-assisted should be interpreted as encompassing diverse computational approaches rather than a single technological category.

In a multicenter, single-blind randomized clinical trial, Ying et al. [16] evaluated an AI-based insulin clinical decision support system (iNCDSS) designed to generate real-time, personalized insulin titration recommendations across multiple subcutaneous insulin regimens in hospitalized adults with poorly controlled T2D. The machine learning-driven system was compared with insulin dose adjustments made by senior endocrinologists and demonstrated noninferior glycemic control over a 5-day intervention period. Mean TIR (70–180 mg/dL) was 76.4% in the iNCDSS group vs. 73.6% in the physician group (treatment difference 2.7%; 95% CI, −2.7% to 8.0%), meeting the predefined noninferiority criterion. Other CGM metrics, including time above or below range, mean sensor glucose, glucose management indicator, and glucose variability, were comparable between groups, while prebreakfast capillary glucose was significantly lower with iNCDSS (131.1 vs. 142.1 mg/dL; *p* = 0.04). Although the AI group received a lower median daily insulin dose (27.2 vs. 30.2 units; *p* = 0.01), this difference was no longer significant after adjustment for baseline insulin requirements, suggesting similar titration intensity. Importantly, no increase in hypoglycemia, severe hypoglycemia, hyperglycemia, ketoacidosis, or other serious adverse events was observed, supporting the safety of AI-guided insulin management. The study is strengthened by its randomized multicenter design, intention-to-treat analysis, integration of the system into routine clinical workflows, and comparison against experienced endocrinologists rather than less intensive standard care. Physician acceptance was notably high, with 98.9% of recommendations adopted and favorable ratings for usability, safety, and time efficiency, indicating potential workflow benefits. Nevertheless, the short follow-up period, conduct within specialized endocrinology wards, and exclusive enrollment of patients from Chinese hospitals limit assessment of long-term effectiveness and broader generalizability. Further research is needed to validate performance in more diverse populations, acute care settings, and outpatient environments, as well as to evaluate sustained clinical outcomes and implementation at scale. Overall, this trial provides robust prospective evidence that AI-assisted insulin titration can achieve glycemic outcomes comparable to expert endocrinologists while maintaining safety and high clinician acceptance, supporting the growing role of AI-enabled decision support in diabetes management. 

In a pilot randomized crossover study, Pryor et al. [17] evaluated AIDANET, a fully closed-loop AID system that replaces the conventional model predictive control algorithm with a highly miniaturized NN. The study randomized six adults with established T1D who completed one week of usual care using commercial hybrid closed-loop systems and one week using AIDANET under free-living conditions. A key innovation was the incorporation of a compact NN requiring only 11 Kb of storage compared with approximately 3500 Kb in the previous version, representing a roughly 300-fold reduction in memory requirements while maintaining near-identical insulin dosing behavior; retrospective analysis showed that 99% of NN-generated microbolus commands deviated by less than 31.7 mU from the original algorithm and no deviations occurred when glucose levels were <70 mg/dL. Glycemic outcomes favored AIDANET, with mean CGM glucose decreasing from 168.0 ± 24.3 mg/dL during usual care to 161.3 ± 16.7 mg/dL and TIR (70–180 mg/dL) increasing, while TAR was reduced and hypoglycemia (time below 70 mg/dL) remained low (1.6% ± 1.8%). Fully closed-loop operation without meal announcements achieved a TIR of 67.7% ± 9.4%, supporting the feasibility of automated insulin dosing independent of carbohydrate counting. The study also explored flexible meal-management approaches; notably, the simplified “Easy Bolus” strategy achieved the highest TIR (70.5%) while maintaining acceptable hypoglycemia exposure (1.5% TBR), suggesting that minimal user input may enhance performance while reducing the burden associated with carbohydrate estimation. Additional strengths include testing under real-world conditions, inclusion of remote safety monitoring, and evaluation of personalization through the POGO scaling module, which enabled management across a broad range of insulin requirements. However, the study was explicitly designed as a safety and feasibility assessment, and the extremely small sample size (n = 6), short duration, homogeneous participant population, absence of statistical hypothesis testing, and limited assessment of patient-reported outcomes substantially restrict conclusions regarding comparative efficacy. The authors also noted that performance during hybrid closed-loop operation may have been affected by conservative insulin settings and uncontrolled dietary variation. Overall, this study provides early clinical evidence that compact NN-based control algorithms can support safe, personalized, and potentially hardware-embedded fully closed-loop insulin delivery, while highlighting the need for larger, adequately powered trials to validate efficacy, compare meal-announcement strategies, and establish long-term effectiveness in more diverse populations. 

In a pilot randomized crossover feasibility trial, Kovatchev et al. [18] introduced the Neural-Net Artificial Pancreas (NAP), a novel neural-network encoding of the established University of Virginia Model Predictive Control (UMPC) algorithm. Adults with T1D who were already using AID systems completed two randomized 20-h hotel-based sessions, receiving either NAP or the original UMPC algorithm in crossover fashion. Glycemic outcomes were comparable, with TIR (70–180 mg/dL) of 86.08% for NAP and 87.25% for UMPC, corresponding to an adjusted difference of only 0.63 percentage points. Similarly, TBR (<70 mg/dL) was 1.88% vs. 1.79%, mean glucose was 130.0 vs. 132.3 mg/dL, and glucose variability remained comparable (coefficient of variation 29.3% vs. 29.1%). Importantly, simultaneous background execution of both algorithms demonstrated that insulin dosing recommendations differed by an average absolute value of only 0.031 U/h under identical inputs, supporting the fidelity of the neural-network approximation. No adverse events or serious safety concerns were observed, and predefined success criteria for differences in TIR, TBR, and TAR were all met. A notable innovation was the generation of the NN from a highly saturated training dataset comprising approximately 4.5 million simulated days of UMPC operation across diverse physiological and behavioral scenarios, enabling replication of complex controller behavior while substantially reducing computational burden. NAP required only 1.4 ms per insulin-dose computation compared with 8.3 ms for UMPC, representing approximately sixfold lower computational demand and eliminating reliance on iterative numerical solvers. Strengths of the study include its randomized crossover design, direct head-to-head comparison with a validated control algorithm, and unique assessment of dosing concordance under identical inputs. However, the study was limited by its small sample size, short intervention duration, controlled hotel environment, and lack of statistical power to formally establish noninferiority. The authors also highlighted broader challenges associated with neural-network “black-box” decision-making, emphasizing the need for rigorous training procedures, external safety supervision, and regulatory oversight. Overall, this study provides an important proof of concept that machine learning-based approximations of validated insulin dosing algorithms can maintain clinical performance, creating a potential pathway for more adaptive, scalable, and resource-efficient AI-driven AID systems.

Unsworth et al. [19] evaluated the Advanced Bolus Calculator for Type 1 Diabetes (ABC4D), an AI-driven decision support system that employs case-based reasoning to adapt and personalize insulin dosing in adults with T1D using multiple daily injections (MDI). In this prospective randomized crossover trial, participants used either ABC4D or a non-adaptive bolus calculator for 12 weeks before crossing over following a 6-week washout period. Despite the theoretical advantage of individualized dose adaptation, ABC4D did not improve glycemic outcomes relative to the comparator, with no significant difference in daytime TIR (3.9–10.0 mmol/L) change between groups (+0.1% [IQR −2.6 to +4.0%] vs. +1.9% [IQR −3.8 to +10.1%]; *p* = 0.53). Importantly, the intervention was found to be safe, with glycemic control maintained at a level comparable to that achieved with the standard calculator. A notable finding was the lower acceptance of AI-generated meal dose recommendations during the intervention phase compared with control (78.7% vs. 93.5%; *p* = 0.009), accompanied by greater reductions from the recommended insulin doses, suggesting that user adherence and trust may have substantially influenced effectiveness. The crossover design and prospective randomization strengthen the internal validity of the study by allowing within-subject comparisons while reducing inter-individual variability. However, the modest sample size, analysis of only 33 participants, relatively short intervention periods, and reliance on participant adherence limit the ability to detect smaller clinical benefits and may constrain generalizability. The findings highlight that the performance of adaptive insulin decision-support systems depends not only on algorithmic accuracy but also on user engagement and acceptance. Future work should focus on improving transparency, usability, and integration of adaptive recommendations into routine diabetes self-management, alongside larger real-world evaluations to determine whether enhanced uptake of AI-generated dosing advice can translate into meaningful glycemic improvements. Overall, this study provides important evidence that AI-based bolus adaptation can be implemented safely, while underscoring the critical role of human factors in realizing the clinical benefits of personalized insulin management.

Nayak et al. [20] investigated the use of a voice-based conversational artificial intelligence (VBAI) system to support autonomous basal insulin titration in adults with T2D requiring initiation or adjustment of once-daily basal insulin. Unlike predictive machine-learning approaches, the intervention combined an Amazon Alexa-based conversational interface with clinician-approved, guideline-based titration algorithms, enabling patients to report fasting glucose values and receive real-time insulin dose adjustments at home. In this randomized clinical trial, the VBAI intervention substantially outperformed standard clinician-managed care across several clinically relevant outcomes. Participants using VBAI achieved optimal insulin dosing significantly faster (median 15 days [IQR 6–27] vs. >56 days [IQR >29.5 to >56]; *p* = 0.006), demonstrated higher insulin adherence (82.9% vs. 50.2%; *p* = 0.01), and were more likely to achieve glycemic control (81.3% vs. 25.0%; *p* = 0.005). Glycemic improvement was also greater, with fasting blood glucose decreasing by 45.9 mg/dL in the VBAI group compared with an increase of 23.0 mg/dL under standard care (difference −68.9 mg/dL; 95% CI −107.1 to −30.7; *p* = 0.001). These benefits appeared to be driven by more frequent insulin titration, as VBAI users received a mean of 7.3 automated dose adjustments compared with 1.6 adjustments in the control group, while maintaining similar healthcare utilization. The intervention additionally reduced diabetes-related emotional distress, reflected by improvements in Problem Areas in Diabetes Scale (PAID-5) scores (difference −3.6 points; 95% CI −6.8 to −0.4; *p* = 0.03), suggesting potential psychosocial as well as metabolic benefits. Safety outcomes were reassuring, with no episodes of severe hypoglycemia, severe hyperglycemia, or adverse events requiring clinician intervention; nonsevere hypoglycemic episodes were autonomously managed through protocol-based dose reductions. A similar number of nonsevere hypoglycemic episodes was reported between groups. Methodological strengths include the randomized design, intent-to-treat analysis, clinician oversight of titration protocols, and evaluation in a decentralized real-world setting. However, interpretation is limited by the small sample size, short 8-week follow-up, reliance on self-reported glucose and adherence data, inclusion of only English-speaking participants, and the use of fasting blood glucose rather than HbA1c to assess glycemic improvement. Furthermore, the study did not compare the voice interface with other digital insulin-titration platforms, making it difficult to isolate the specific contribution of conversational AI. Overall, this trial provides early evidence that AI-enabled conversational systems may accelerate insulin dose optimization and improve adherence in T2D, highlighting the potential of patient-facing AI tools to address therapeutic inertia while underscoring the need for larger, longer-term studies evaluating durability, scalability, and integration into routine diabetes care.

Jacobs et al. [21] evaluated an exercise-aware model predictive control (exMPC) AID system that incorporates real-time metabolic expenditure estimates derived from smartwatch heart rate and accelerometry data to automatically adjust insulin delivery and includes a long short-term memory (LSTM)-based predictive low-glucose suspend safety layer. In a randomized, non-blinded crossover trial involving adults with T1D (mean age 34.4 years; HbA1c 6.4%), exMPC was compared with an exercise-aware adaptive proportional derivative (exAPD) system that shared the same LSTM-based safety layer but required user confirmation of detected exercise and applied predefined insulin reduction actions. The primary endpoint, TBR (<3.9 mmol/L) during a 12-h in-clinic session, did not differ significantly between systems (1.3% vs. 2.5%, *p* = 0.46). However, in the 2-h post-exercise window, exMPC achieved significantly lower mean glucose (7.3 vs. 8.0 mmol/L, *p* = 0.023) with comparable TBR. Across the full 76-h study period, both systems achieved clinically acceptable glycemic outcomes, with TIR exceeding 70% (71.2% vs. 75.5%) and similarly low TBR (0.96% vs. 1.30%), and no adverse events reported. The study’s strengths include its crossover design, integration of real-world wearable sensor data into AID systems, and evaluation across both controlled and free-living conditions, supporting feasibility of continuous physical activity-aware insulin modulation. Key limitations include the small sample size, short duration, and limited generalizability given a relatively young, well-controlled cohort and reliance on a single type of wearable device, alongside insufficient power to detect small between-system differences. Additionally, the short structured exercise exposure and procedural heterogeneity introduced by staged safety rollouts may have influenced outcome variability. Overall, this study demonstrates that continuous integration of wearable-derived exercise metrics into AID systems is feasible and can maintain safe glycemic control, while suggesting potential advantages of fully automated activity-responsive algorithms in refining post-exercise glucose stability and reducing user burden in T1D management.

Avari et al. [22] conducted a multicentre, open-label, crossover randomized controlled trial evaluating the Patient Empowerment through Predictive Personalized Decision Support (PEPPER) system, an AI-enabled adaptive insulin bolus advisor incorporating case-based reasoning alongside a predictive safety framework including predictive glucose alerts and alarms, predictive low-glucose suspend, and personalized carbohydrate recommendations. The study included adults with T1D treated with MDI or insulin pump therapy and compared PEPPER with a standard bolus calculator across two 12-week intervention periods separated by washout. The primary outcome, percentage TIR (3.9–10.0 mmol/L), did not differ significantly between PEPPER and control (62.5% vs. 58.4%, *p* = 0.27), and no significant differences were observed across secondary glycemic endpoints including hypoglycemia, hyperglycemia, glycemic variability indices, postprandial outcomes, or HbA1c. Despite the absence of statistically significant glycemic benefit, the system demonstrated operational feasibility, processing 14,723 meal bolus cases with a median of 4.0 (3.3–5.5) cases per participant per day, with 87 (65–96)% of recommended boluses accepted. Safety analysis indicated no increase in adverse glycemic exposure, with 5 (2–14)% of bolus recommendations constrained by safety limits and no significant differences in alerts, alarms, or insulin delivery interruptions between study arms. Methodologically, the crossover design and inclusion of both MDI and pump users strengthen within-participant comparability and generalizability, although interpretation is limited by early study termination due to device discontinuation, modest sample size, and high attrition, which likely reduced statistical power. Additionally, participants exhibited relatively moderate baseline glycemic control, potentially limiting measurable incremental benefit, and the system’s dependence on manual case revision and multi-device integration may hinder scalability in routine care. The authors highlight the need for fully automated case-base updating, simplified hardware integration, and larger, adequately powered trials of sufficient duration to assess clinical effectiveness. Overall, this study contributes important randomized evidence that an AI-driven adaptive bolus calculator with integrated safety systems can be safely implemented in both MDI and pump users, but further optimization and adequately powered trials are required to determine its impact on glycemic outcomes in routine diabetes management.

Across the included trials, the clinical impact of AI-assisted insulin management appears to depend less on the presence of AI alone and more on the level of automation, the quality of input data, the clinical context, and the degree of interaction required from users. Systems that directly automated insulin adjustment or provided frequent, data-driven titration support, such as closed-loop insulin delivery platforms and conversational AI-guided basal insulin titration, demonstrated improvements in selected outcomes. In contrast, adaptive decision-support tools that relied on patient acceptance of AI-generated recommendations showed more variable effects, suggesting that algorithmic accuracy does not necessarily translate into clinical benefit when implementation depends heavily on user behavior. These findings indicate that future evaluations should consider AI systems as integrated socio-technical interventions rather than isolated algorithms.

Importantly, the available evidence does not demonstrate that AI consistently outperforms expert clinical decision-making or established diabetes technologies. Instead, most randomized trials suggest that AI can achieve outcomes comparable to current standards while potentially reducing treatment burden, increasing dose-adjustment frequency, and improving personalization. The absence of clear superiority across many studies may reflect several factors, including limited sample sizes, relatively well-controlled study populations, short follow-up periods, and ceiling effects among participants already using advanced diabetes technologies. Therefore, the current evidence supports AI primarily as an augmentation strategy that may enhance scalability and personalization rather than as a replacement for clinical expertise.

A critical challenge emerging from these studies is the distinction between technical validation and clinical effectiveness. Some neural-network-based systems demonstrated excellent agreement with reference algorithms and substantial computational advantages, but these findings represent validation of algorithmic performance rather than proof of improved patient outcomes. Conversely, systems that showed improvements often incorporated additional elements, such as structured feedback, increased monitoring frequency, improved adherence, or simplified patient interactions. Future research should therefore evaluate not only whether AI predictions or recommendations are accurate, but also whether their implementation produces sustained improvements in glycemic control, quality of life, healthcare efficiency, and equitable access across diverse populations.

The heterogeneity of AI approaches included in this review also highlights that “AI-assisted insulin management” represents a broad spectrum of technologies rather than a single therapeutic intervention. Differences in algorithm architecture, degree of automation, required user input, and clinical workflow integration may influence both effectiveness and scalability. Consequently, direct comparisons between AI systems should be interpreted cautiously, and future trials should incorporate standardized outcome measures, longer follow-up, diverse patient populations, and implementation endpoints to determine which characteristics of AI systems provide meaningful clinical value.

Overall, the available evidence suggests that AI-assisted insulin management systems can be implemented safely within the settings evaluated and may achieve glycemic outcomes broadly comparable to standard care or expert-guided insulin management. However, these findings should be interpreted cautiously because they are based on a limited number of heterogeneous RCTs, some of which were pilot or feasibility studies with small sample sizes and short follow-up periods. Furthermore, only a subset of studies contributed data for each clinical outcome, limiting confidence in the consistency of the observed effects. Although some interventions—particularly AID systems and conversational AI-supported insulin titration—showed improvements in selected glycemic and implementation outcomes, the evidence remains insufficient to determine whether these benefits are generalizable across AI platforms or patient populations. Consequently, the current evidence supports further investigation rather than definitive conclusions regarding the comparative effectiveness of AI-assisted insulin management.

Among studies restricted to individuals with T1D, AI-assisted insulin management systems demonstrated consistent evidence of safety and feasibility but variable evidence of incremental glycemic benefit. Fully automated or AI-enhanced AID approaches generally maintained low hypoglycemia exposure. Neural-network-based controllers, including AIDANET and the NAP, demonstrated comparable performance to original algorithms while reducing computational requirements, supporting their potential for implementation in resource-constrained devices. Similarly, the exMPC system demonstrated feasibility of incorporating wearable-derived activity data into insulin delivery decisions, with improvements in post-exercise glucose control but no significant reduction in exercise-related hypoglycemia compared with the comparator system. In contrast, AI-based decision-support systems requiring active user acceptance, such as ABC4D and PEPPER, were safe and feasible but did not demonstrate significant improvements in TIR compared with conventional bolus calculators. These findings suggest that within T1D, the clinical impact of AI systems may depend on the degree of automation, integration of real-time physiological data, and the extent to which effectiveness relies on patient interaction with AI recommendations.

Table 2 summarizes the findings across the included RCTs according to the main outcome domains evaluated. The studies are grouped by glycemic efficacy, insulin titration and dosing, safety, and implementation-related outcomes, providing an overview of the consistency and heterogeneity of the evidence across different AI-assisted insulin management systems.

To further explore the clinical heterogeneity identified in this review, the included studies were considered according to diabetes type and AI technology category. Among individuals with T1D, most investigated systems were designed for AID or personalized bolus decision support, reflecting the intensive insulin management requirements of this population. AI-enhanced AID approaches, including neural-network controllers (AIDANET and NAP) and exMPC systems, consistently demonstrated safety, and generally maintained favorable glycemic outcomes. However, these studies primarily evaluated algorithmic feasibility, controller equivalence, or short-term performance rather than superiority over established AID technologies. In contrast, AI-based decision-support systems requiring active patient engagement, such as ABC4D and PEPPER, demonstrated feasibility and safety but did not produce significant improvements in glycemic outcomes compared with conventional bolus calculation approaches. This difference suggests that in T1D, the clinical benefit of AI may be influenced by the level of automation, integration of real-time physiological data, and the extent to which outcomes rely on sustained user interaction.

The two studies conducted in adults with T2D evaluated substantially different clinical scenarios and AI applications compared with the T1D studies. The iNCDSS trial evaluated inpatient insulin titration support using clinician-facing recommendations, demonstrating noninferior glycemic control compared with experienced endocrinologists in a supervised hospital setting. Conversely, the voice-based conversational AI intervention targeted outpatient basal insulin management and appeared to improve insulin titration efficiency and adherence compared with standard care. These findings indicate that in T2D, where insulin initiation and titration are frequently limited by therapeutic inertia and healthcare access barriers, AI may provide benefit primarily through decision support, workflow optimization, and increased treatment engagement rather than through fully AID.

Differences in AI architecture also appeared to influence observed outcomes. Closed-loop and adaptive control systems that directly modified insulin delivery generally demonstrated consistent safety and maintained or modestly improved glycemic metrics, whereas recommendation-based systems showed more variable effectiveness, partly because clinical benefit depended on patient acceptance of AI-generated suggestions. Neural-network approaches demonstrated computational advantages and algorithmic fidelity but were not designed to establish clinical superiority. Conversely, conversational AI systems produced clinically meaningful improvements in selected implementation outcomes, although their effects may reflect increased monitoring frequency and engagement in addition to the AI component itself. Therefore, the term “AI-assisted insulin management” encompasses distinct technological strategies with different mechanisms of action, target populations, and expected clinical benefits, and future studies should avoid treating these approaches as a single intervention category.

### 4.1. Strengths and Limitations of the Evidence

The current body of randomized evidence evaluating AI-assisted insulin management demonstrates several important strengths that support the robustness and clinical relevance of the findings. First, all included studies were RCTs, representing a high level of evidence for intervention efficacy and safety in clinical research. This is particularly relevant in the context of AI-driven insulin delivery systems, where algorithmic recommendations directly influence medication dosing and, consequently, patient safety [23]. The inclusion of both parallel-group and crossover designs also allowed for complementary methodological advantages, with crossover trials reducing inter-individual variability and parallel trials supporting between-group comparisons in more heterogeneous clinical settings [24].

A second strength lies in the consistent use of objective, high-resolution glycemic outcome measures, particularly CGM-derived metrics such as TIR, TBR, and glycemic variability. These endpoints provide granular and clinically meaningful assessments of glycemic control, improving comparability across studies and reducing reliance on surrogate or self-reported measures. In addition, several trials incorporated safety endpoints and implementation-related outcomes, including hypoglycemia rates, insulin dosing behavior, adherence, and user acceptance, allowing for a more comprehensive evaluation of AI systems beyond purely metabolic outcomes.

Another important strength is the diversity of AI architectures and clinical applications represented across the included trials. The evidence base spans decision-support systems, adaptive bolus calculators, conversational AI platforms, and fully closed-loop AID systems incorporating machine learning and NN approaches. This heterogeneity, while methodologically challenging for synthesis, provides a broad perspective on the current landscape of AI-enabled insulin management and allows for preliminary identification of patterns related to system design, level of automation, and clinical effectiveness. Additionally, the inclusion of both type 1 and type 2 diabetes populations and multiple healthcare settings (inpatient, outpatient, and free-living environments) enhances the external relevance of the findings.

Despite these strengths, several important limitations affect the certainty and generalizability of the evidence. Overall, the evidence base is characterized by relatively small sample sizes, short intervention durations, and several pilot or feasibility studies. Some trials were not primarily powered to detect clinically meaningful differences in glycemic outcomes, limiting the ability to draw definitive conclusions regarding comparative effectiveness. Short follow-up periods further restrict assessment of long-term glycemic control, sustainability of algorithm performance, and potential impacts on diabetes-related complications. Importantly, the limited number of available RCTs (n = 7) restricts the statistical certainty of the conclusions and prevents robust assessment of whether observed benefits are consistent across different diabetes populations, healthcare settings, and AI technologies.

Although quantitative effect estimates were available for individual trials, meta-analysis was considered inappropriate because the included interventions did not represent a sufficiently comparable clinical question. The variability in AI architecture, degree of automation, diabetes population, comparator interventions, follow-up duration, and outcome definitions would have resulted in pooled estimates with limited clinical interpretability.

In addition, there is substantial heterogeneity in intervention design, degree of automation, patient populations, and outcome reporting. This variability reflects the early developmental stage of AI-assisted insulin technologies but precludes quantitative synthesis and limits direct comparability across studies. Differences in baseline glycemic control, prior experience with insulin technology, and study environments (controlled inpatient settings vs. real-world free-living conditions) further contribute to variability in observed effects. Although subgroup interpretation by diabetes type and AI modality provided important contextual insights, formal subgroup analyses were limited by the small number of available randomized trials within each category. The available evidence therefore supports hypothesis generation regarding differential effects of AI systems in type 1 vs. type 2 diabetes and across technological approaches, but does not allow definitive conclusions regarding comparative effectiveness between AI modalities.

Another key limitation relates to human–AI interaction factors, which were inconsistently measured and reported. Several studies indicated that user adherence, trust, and acceptance of AI-generated recommendations influenced effectiveness, particularly for decision-support systems. However, the lack of standardized assessment of behavioral and implementation outcomes limits understanding of how these factors mediate clinical performance in real-world practice. Similarly, transparency and interpretability of AI algorithms were rarely addressed in a systematic manner, despite their relevance for clinical adoption and regulatory evaluation.

Risk of bias was low in only one study, with the remaining trials rated as having some concerns due to attrition, early termination, or other methodological limitations. Although objective CGM-based outcomes reduce the likelihood of measurement bias, methodological constraints such as incomplete reporting of randomization or allocation concealment and missing outcome data may have influenced effect estimates. Formal assessment of publication bias was not performed because meta-analysis was not conducted and the number of available RCTs was insufficient for reliable funnel plot interpretation or statistical testing. Given the substantial heterogeneity in AI interventions, patient populations, comparators, and reported outcomes, publication bias assessment based on pooled estimates was considered inappropriate.

An additional limitation of this review is that relatively few studies contributed to each outcome domain. Although seven RCTs met the eligibility criteria, not all studies reported the same glycemic, safety, or implementation outcomes, and outcome definitions were not always consistent across trials. Consequently, the evidence supporting individual outcomes should be interpreted with caution, as conclusions are often based on a limited number of studies.

Finally, external validity remains limited. Several studies enrolled highly selected populations, often consisting of technologically experienced individuals with relatively well-controlled diabetes, which may not reflect broader real-world populations with varying levels of digital literacy, comorbidities, and access to diabetes technologies. Furthermore, most trials were conducted in high-resource healthcare settings, limiting generalizability to low- and middle-income contexts where implementation challenges may differ substantially.

Collectively, while the evidence base provides reassuring signals regarding safety and feasibility of AI-assisted insulin management, the certainty of evidence regarding comparative effectiveness remains limited. Future high-quality, adequately powered randomized trials with longer follow-up and standardized outcome frameworks are essential to strengthen the evidence base and support translation into routine clinical practice.

**Figure 2:**
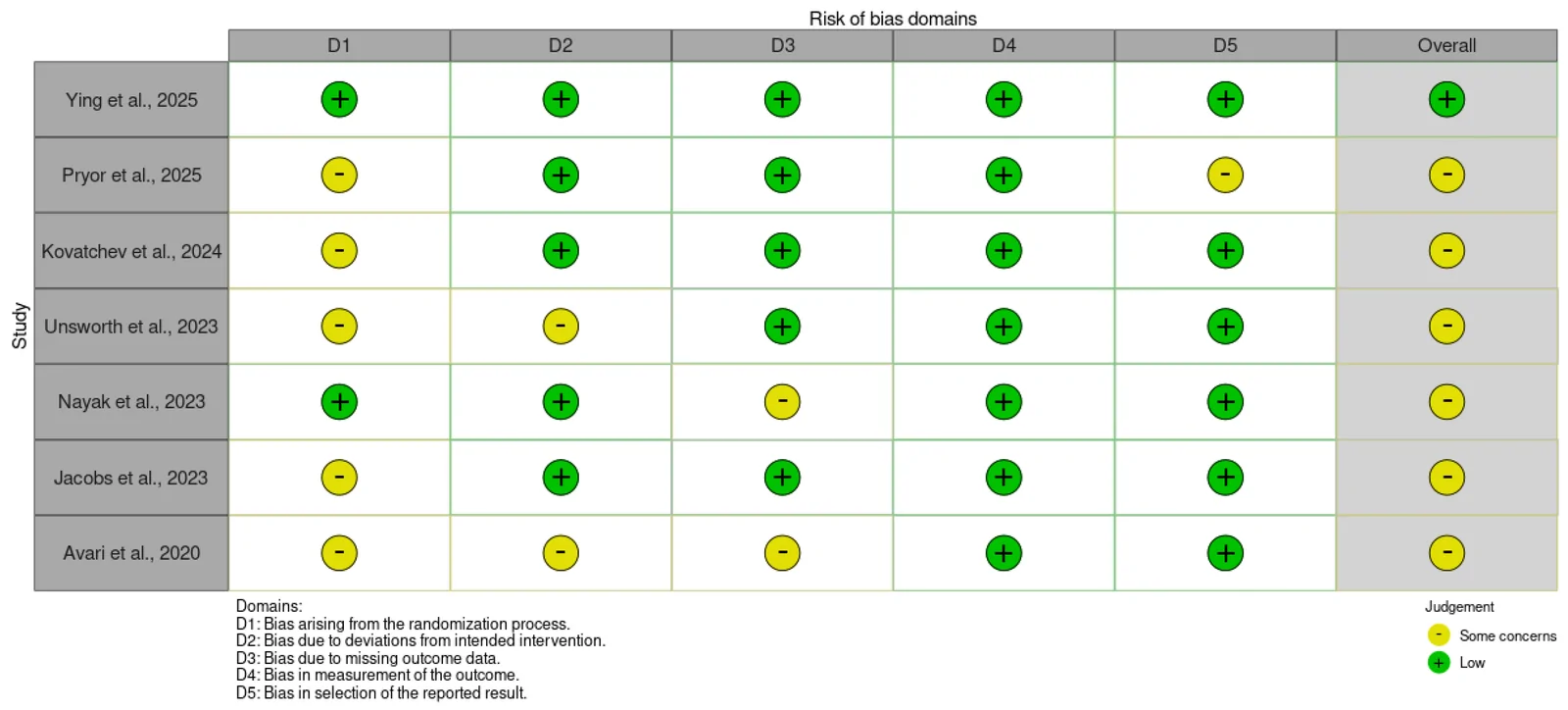
Risk of bias (RoB) assessment of the included studies [15,16,17,18,19,20,21,22].

The risk of bias of the included studies was assessed using the Risk of Bias (RoB) tool, considering the domains of randomization process, deviations from intended interventions, missing outcome data, measurement of outcomes, and selection of the reported result (Figure 2). The risk of bias assessments were further supported by detailed qualitative justifications for each domain across the included RCTs evaluating AI-assisted insulin management systems in diabetes (Table 3).

**Figure 3:**
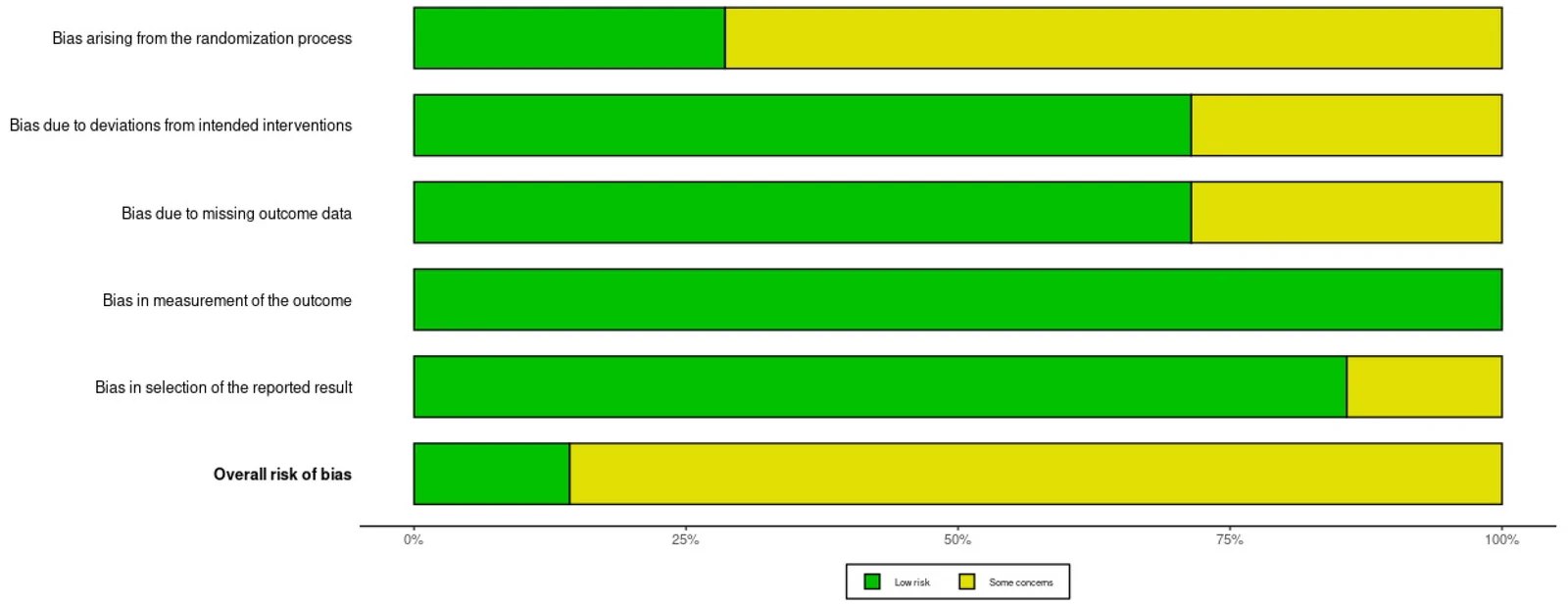
Risk of bias summary across the included randomized controlled trials evaluating artificial intelligence-assisted insulin management systems [15].

The overall risk of bias assessment demonstrated that methodological quality varied across the included studies (Figure 3). Most studies showed low risk of bias in outcome measurement and selection of reported results, reflecting the use of objective glycemic outcomes, particularly CGM-derived measures. However, several studies were judged to have some concerns, mainly due to potential deviations from intended interventions, limited reporting of randomization procedures, attrition, and early study termination.

### 4.2. Clinical and Implementation Implications

**Figure 4:**
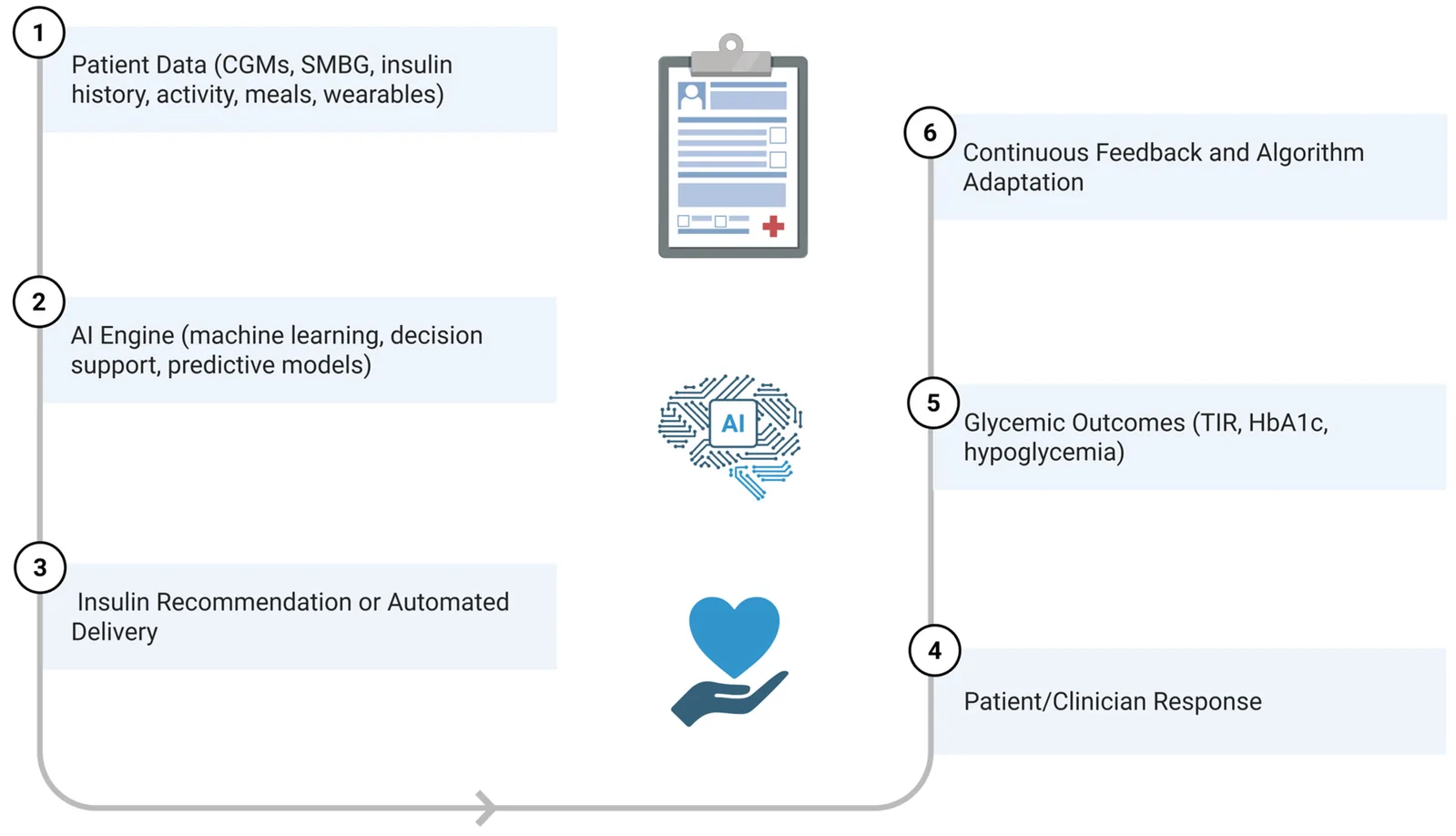
Conceptual framework of AI-assisted insulin management systems. Patient data derived from continuous glucose monitoring (CGM), self-monitoring of blood glucose (SMBG), insulin administration records, physical activity, meal intake, and wearable devices are integrated into an AI engine that utilizes machine learning, predictive modeling, and clinical decision-support algorithms. The system generates insulin dosing recommendations or enables automated insulin delivery, influencing patient and clinician responses. These actions affect glycemic outcomes, including time in range (TIR), glycated hemoglobin (HbA1c), and hypoglycemia. Outcome data are continuously fed back into the system to support ongoing algorithm adaptation and performance optimization. Created in BioRender (https://biorender.com/r6164tt), last accessed on 24 July 2026.

Figure 4 presents the conceptual framework of AI-assisted insulin management systems. Patient-generated and clinical data, including CGM, self-monitoring of blood glucose (SMBG), insulin administration history, physical activity, meal intake, and wearable device data, are processed by an AI engine employing machine learning, predictive analytics, and decision-support algorithms. The AI system generates insulin dosing recommendations or facilitates AID, which subsequently informs patient and clinician actions. These interventions influence glycemic outcomes, including TIR, HbA1c, and hypoglycemic events. Continuous feedback from patient outcomes is then used to refine and adapt algorithm performance over time.

Although the current evidence remains preliminary, it provides insights into the potential clinical implementation of AI-assisted insulin management systems. Collectively, the included trials suggest a trend toward increasing automation in insulin management; however, this observation is based on a limited number of heterogeneous studies and should therefore be interpreted cautiously. This evolution is most evident in closed-loop AID systems and conversational AI platforms, which increasingly reduce the need for manual insulin titration while maintaining clinician-defined safety boundaries.

Despite this technological progression, the available evidence suggests that human oversight remains an essential component of safe and effective AI-assisted insulin therapy. Even in highly automated systems, clinicians are required to define safety constraints, validate algorithmic performance, and manage complex clinical scenarios that fall outside predefined decision rules. Similarly, patient involvement remains critical in many interventions, particularly those relying on MDI or adaptive bolus calculators, where insulin delivery is still partially dependent on user behavior.

Several studies suggested that user adherence, trust, and engagement with AI-generated recommendations are major determinants of clinical effectiveness. Interventions with high acceptance of AI-driven dosing suggestions tended to demonstrate more favorable glycemic outcomes, whereas reduced adherence or selective overriding of recommendations attenuated potential benefits. This highlights that the performance of AI systems cannot be fully understood in isolation from behavioral and psychological factors, reinforcing the importance of human–AI interaction as a central determinant of therapeutic success.

In this context, explainability and transparency emerge as critical barriers to widespread clinical adoption. Several AI-based insulin management systems, particularly those based on NN or complex adaptive algorithms, operate as partially opaque “black-box” models, limiting interpretability for both clinicians and patients. This lack of transparency may affect trust, hinder clinical validation, and complicate regulatory approval processes. Consequently, future development of AI systems in diabetes care will likely need to balance predictive performance with interpretability and clinical comprehensibility.

From a regulatory perspective, the dynamic and adaptive nature of AI-driven insulin systems presents additional challenges. Unlike static medical devices, many of these systems are capable of continuous learning or algorithmic updates, raising questions regarding post-market surveillance, version control, and ongoing safety validation. Regulatory frameworks for such adaptive systems are still evolving, and harmonized international standards will be required to ensure safe deployment while enabling innovation.

Another important implication relates to system integration within broader digital health ecosystems. The most promising interventions identified in this review generally incorporated real-time data streams from CGM, wearable sensors, and digital health platforms. Future clinical impact is likely to depend on the degree to which AI systems can be seamlessly integrated with electronic health records, patient-facing mobile applications, and multi-modal physiological monitoring devices. Such integration would enable more comprehensive and context-aware insulin decision-making, potentially improving both precision and responsiveness of therapy.

Overall, the evidence suggests that the future success of AI-assisted insulin management will depend not only on algorithmic sophistication but also on its ability to be embedded within clinically usable, transparent, and patient-centered care pathways. The transition from experimental systems to routine clinical tools will require careful attention to human factors, regulatory alignment, interoperability, and long-term safety monitoring.

## 5. Conclusions and Recommendations for Future Studies

This systematic review synthesized evidence from seven RCTs evaluating AI-assisted insulin management systems in individuals with type 1 and type 2 diabetes. Although the included studies investigated a broad range of AI technologies, the evidence base remains limited and heterogeneous with respect to intervention design, study populations, clinical settings, and reported outcomes. Consequently, the findings should be interpreted as preliminary.

Across the included studies, AI-assisted insulin management systems generally demonstrated favorable safety profiles and achieved glycemic outcomes that were comparable to standard care in the evaluated settings. However, these findings are based on a small number of heterogeneous studies, and the certainty of evidence regarding comparative effectiveness remains limited. Across both inpatient and outpatient settings, no study reported severe hypoglycemia, diabetic ketoacidosis, or other serious adverse events attributable to AI-assisted insulin dosing. These findings suggest that contemporary AI-based insulin management systems may be implemented safely when accompanied by appropriate safeguards, monitoring mechanisms, and clinical oversight.

Some studies reported improvements in insulin titration efficiency, selected glycemic outcomes, or implementation-related measures, particularly among more highly automated systems. However, these findings were not consistent across interventions, and evidence for each outcome was often derived from only a few studies. Systems designed to automate insulin delivery or provide frequent real-time dose adjustments generally showed promising results. In particular, fully closed-loop neural-network-based insulin delivery systems and conversational AI-guided basal insulin titration demonstrated improvements in glycemic outcomes, treatment adherence, and insulin optimization while reducing user burden. Conversely, adaptive bolus calculators and case-based decision-support tools frequently achieved glycemic outcomes comparable to standard approaches but did not consistently produce statistically significant improvements in TIR or other glycemic metrics. These findings indicate that the effectiveness of AI technologies depends not only on algorithmic sophistication but also on the degree to which they can reduce therapeutic inertia, respond dynamically to physiological changes, and support real-world patient behavior.

An important observation across multiple studies was the influence of human factors on clinical effectiveness. Several interventions relied on user acceptance of AI-generated recommendations, and lower adherence to suggested insulin doses appeared to attenuate potential benefits. This highlights that successful implementation of AI-assisted insulin management requires more than accurate algorithms; transparency, usability, trust, and integration into routine workflows are likely to be critical determinants of effectiveness. The findings therefore support a model of human-centered AI in which technology augments rather than replaces patient and clinician decision-making.

The review also highlights important limitations within the current evidence base. Several studies were pilot or feasibility trials, and many had relatively small sample sizes, short follow-up periods, and highly selected participant populations. The limited duration of follow-up also restricted the assessment of long-term clinical efficacy, including the durability of glycemic improvements, sustained treatment adherence, long-term safety, and potential effects on diabetes-related complications. Larger, adequately powered RCTs with standardized outcome reporting and longer follow-up are needed to establish the durability of glycemic benefits, long-term clinical efficacy, safety, and generalizability of AI-assisted insulin management across diverse patient populations. Extended follow-up periods are particularly important to determine whether short-term improvements in glycemic control are sustained over time and to evaluate long-term outcomes, including treatment adherence, patient engagement, insulin dose optimization, quality of life, healthcare utilization, and potential effects on diabetes-related complications.

Many studies enrolled individuals who were already experienced with diabetes technologies or who had relatively well-controlled diabetes, potentially limiting the ability to detect clinically meaningful improvements and reducing generalizability to broader populations. Furthermore, substantial heterogeneity in intervention characteristics and outcome reporting prevented quantitative synthesis and made direct comparisons between AI approaches difficult.

Future research should focus on large-scale, adequately powered randomized trials with longer follow-up periods to determine the durability of glycemic benefits, safety, and patient engagement over time. Studies should include more diverse populations across different age groups, ethnic backgrounds, healthcare systems, and levels of digital literacy to improve external validity. Standardization of outcome reporting, particularly for CGM metrics, insulin dosing outcomes, patient-reported outcomes, and implementation measures, would facilitate comparisons across studies and support future meta-analyses. Further investigation is also needed to evaluate the impact of AI systems on healthcare utilization, clinician workload, cost-effectiveness, and long-term diabetes complications.

In addition, future development should prioritize explainable and transparent AI approaches that enhance user trust and facilitate clinical oversight. Greater integration of multimodal data streams, including CGM, wearable sensor data, physical activity, nutrition, and behavioral inputs, may further improve personalization and responsiveness of insulin management systems. Research should also address regulatory, ethical, and cybersecurity considerations associated with increasingly autonomous insulin dosing technologies. Given that the available evidence is derived from only seven RCTs, conclusions regarding comparative effectiveness, long-term clinical benefit, and widespread implementation should remain cautious.

In conclusion, the available randomized evidence suggests that AI-assisted insulin management systems are feasible and appear to have acceptable short-term safety within the settings studied. Some technologies demonstrated improvements in selected glycemic and implementation outcomes; however, the evidence remains limited by the small number of randomized trials, heterogeneity in interventions and reported outcomes, and generally short follow-up periods. Accordingly, definitive conclusions regarding comparative effectiveness and routine clinical implementation cannot yet be drawn. Larger, adequately powered RCTs with standardized outcome reporting and longer follow-up are needed to establish the long-term efficacy, safety, and generalizability of AI-assisted insulin management across diverse patient populations.

## Figures and Tables

**Table 1:** Characteristics of randomized controlled trials evaluating artificial intelligence-assisted insulin management in diabetes.

Study	Country	Population (Randomized)	Diabetes Type	Intervention (AI System)	Comparator	Duration	Primary Outcomes	Main Findings
Ying et al., 2025 [16]	China	149 hospitalized adults with poorly controlled diabetes (mean age 64.2 years; mean HbA1c 9%) receiving insulin therapy in endocrinology wards	T2D	AI-based iNCDSS providing real-time personalized insulin titration recommendations	Insulin titration by senior endocrinology physicians (>10 years of experience)	5 days	Percentage of time in target glucose range (TIR 70–180 mg/dL) measured by CGM during the intervention period	iNCDSS achieved noninferior glycemic control compared with senior physicians (TIR: 76.4% vs. 73.6%; treatment difference 2.7%, 95% CI −2.7% to 8.0%), with no increase in hypoglycemia or adverse events. Physicians showed high acceptance (98.9% recommendation adherence) and reported high satisfaction with the system.
Pryor et al., 2025 [17]	United States	6 adults (18–60 years; diabetes duration 24.9 ± 8.7 years; HbA1c 7.17 ± 0.41%) with established T1D using insulin pumps and CGM in free-living conditions	T1D	AIDANET: fully closed-loop AID system using a miniaturized NN to replace model predictive control, with optional Easy Bolus and carbohydrate-counting modes	Participants’ usual care (commercial hybrid closed-loop AID systems) in a randomized crossover design	7 days usual care vs. 7 days AIDANET (including 5 fully closed-loop days, 1 Easy Bolus day, and 1 carbohydrate-counting day)	Mean CGM glucose and Glucose Management Indicator; secondary outcomes included TIR (70–180 mg/dL), TBR, TAR, and glycemic variability (CGM coefficient of variation)	AIDANET demonstrated feasibility and safety with no serious adverse events. Compared with usual care, mean glucose improved (161.3 vs. 168.0 mg/dL) and TIR increased. Fully closed-loop operation achieved safe glycemic control without meal announcements. Easy Bolus mode showed the highest TIR (70.5%). The miniaturized NN reduced memory requirements by ~300-fold while maintaining performance comparable to the original algorithm.
Kovatchev et al., 2024 [18]	United States	15 adults with established T1D already using AID (mean age 47.9 years; mean diabetes duration 26.1 years; HbA1c 5.4–8.1%)	T1D	NAP: neural-network approximation of the UMPC algorithm	UMPC algorithm	Two crossover hotel sessions of 20 h each	Difference in TIR (70–180 mg/dL) between NAP and UMPC	NAP achieved glycemic control comparable to that of UMPC (TIR 86.08% vs. 87.25%). Insulin recommendations differed by only 0.031 U/h on average. No serious adverse events occurred. NAP required approximately sixfold less computational effort than UMPC.
Unsworth et al., 2023 [19]	United Kingdom	37 adults on MDI of insulin (median age 44.7 years, diabetes duration 15.0 years, HbA1c 61 mmol/mol)	T1D	ABC4D using case-based reasoning adaptive insulin bolus decision support (smartphone app + web portal)	Non-adaptive bolus calculator	Two 12-week intervention periods with washout (crossover)	Difference in % TIR (3.9–10.0 mmol/L) during daytime (07:00–22:00) between groups	No significant difference in daytime % TIR change with ABC4D compared with control (*p* = 0.53). Lower acceptance of meal dose recommendations in ABC4D arm, with greater deviation from suggested insulin doses, likely limiting effectiveness. System considered safe but no glycemic improvement vs. control.
Nayak et al., 2023 [20]	United States	39 adults with T2D requiring initiation or adjustment of once-daily basal insulin	T2D	VBAI application using an Amazon Alexa-based conversational interface to autonomously guide basal insulin titration according to clinician-approved protocols	Standard of care basal insulin management by clinicians with daily online insulin/glucose logging	8 weeks	Time to optimal insulin dose; insulin adherence; changes in composite diabetes-related emotional distress scores, attitudes toward health technology, and medication adherence	VBAI significantly reduced time to optimal insulin dose (median 15 days vs. >56 days; *p* = 0.006), improved insulin adherence (82.9% vs. 50.2%; *p* = 0.01), increased achievement of glycemic control (81.3% vs. 25.0%; *p* = 0.005), reduced fasting blood glucose (−45.9 vs. +23.0 mg/dL; *p* = 0.001), and decreased diabetes-related emotional distress. No severe hypoglycemia, hyperglycemia, or adverse events occurred.
Jacobs et al., 2023 [21]	United States	25 adults with T1D using insulin pumps (mean age 34.4 years; mean HbA1c 6.4%; mean diabetes duration 22.8 years)	T1D	AI-augmented exMPC AID system integrating smartwatch-derived metabolic expenditure information (heart rate, accelerometry/METs) to automatically adjust insulin	AI-augmented exAPD AID system requiring user-confirmed exercise detection and predefined insulin reduction actions	Two 76-h (12-h in-clinic session plus 2 free-living days) intervention periods with washout (crossover)	Primary: % TBR (<3.9 mmol/L) during 12-h in-clinic session. Secondary: % TIR (3.9–10 mmol/L), mean glucose, % TAR, rescue carbohydrates, insulin use, % time glucose was very low (˂3 mmol/L) or very high (>13.9 mmol/L).	exMPC and exAPD achieved comparable glucose outcomes. Primary endpoint was not significantly different (TBR 1.3% vs. 2.5%, *p* = 0.46). However, during the 2 h after the start of structured exercise, exMPC produced significantly lower mean glucose (7.3 vs. 8.0 mmol/L, *p* = 0.023), without a significant difference in TBR. The study demonstrated that wearable fitness sensor data could be integrated into AID systems to automate insulin adjustments during exercise and daily activities.
Avari et al., 2020 [22]	United Kingdom, Spain	50 adults on MDI or insulin pump	T1D	PEPPER adaptive insulin recommender system	Standard bolus calculator	Two 12-week intervention periods with washout (crossover)	% TIR (3.9–10.0 mmol/L)	No significant difference in TIR between PEPPER vs. control (62.5% vs. 58.4%, *p* = 0.27); system was safe and feasible but did not significantly improve glycemic outcomes vs. standard bolus calculator. No objective difference in time spent in hypoglycemia.

Abbreviations: ABC4D, Advanced Bolus Calculator for Type 1 Diabetes; AI, Artificial Intelligence; AID, Automated Insulin Delivery; CGM, Continuous Glucose Monitoring; exAPD, Exercise-Aware Adaptive Proportional Derivative; exMPC, Exercise-Aware Model Predictive Control; HbA1c, Glycated Hemoglobin; iNCDSS, Insulin Clinical Decision Support System; MDI, Multiple Daily Injections; NAP, Neural-Net Artificial Pancreas; NN, Neural Networks; PEPPER, Patient Empowerment through Predictive Personalized Decision Support; T1D, Type 1 Diabetes; T2D, Type 2 Diabetes; TAR, Time Above Range; TBR, Time Below Range; TIR, Time in Range; UMPC, University of Virginia Model Predictive Control; VBAI, Voice-Based Conversational Artificial Intelligence.

**Table 2:** Summary of outcomes reported across the included randomized controlled trials evaluating AI-assisted insulin management systems in diabetes.

Outcome	Studies	Overall Findings
Time in Range (TIR)	[16,17,18,19,21,22]	AI systems generally achieved glycemic control comparable to standard care, with modest improvements in some closed-loop systems.
Time Below Range (TBR)	[16,17,18,19,21,22]	No clear increase in hypoglycemia; TBR was generally similar between intervention and comparator groups.
Glucose levels	[16,17,18,19,20,21,22]	Improvements in mean glucose or fasting glucose in some studies, with comparable glycemic outcomes in others.
Insulin dose/titration	[16,20]	AI facilitated efficient insulin titration while maintaining safety.
Safety	[16,17,18,19,20,21,22]	No increase in severe hypoglycemia or serious adverse events across studies.
Implementation outcomes	[16,19,20,22]	High clinician acceptance and improved adherence in some studies, although acceptance of AI recommendations varied; feasibility for routine-care integration.

**Table 3:** Justification of risk of bias assessments for included randomized controlled trials evaluating AI-assisted insulin management systems in diabetes.

Study	Risk of Bias Assessment Justification
Ying et al., 2025 [16]	The methodological quality of the trial is high, with appropriate randomization, intention-to-treat analysis, a low dropout rate, an objective outcome measure, and a prospectively registered protocol.
Pryor et al., 2025 [17]	The study was assessed as having some concerns due to insufficient reporting of the randomization process and incomplete reporting of prespecified outcomes.
Kovatchev et al., 2024 [18]	The study was assessed as having some concerns due to inadequate reporting of randomization process.
Unsworth et al., 2023 [19]	The study was judged to have some concerns due to insufficient detail regarding the randomization and allocation concealment processes, as well as potential concerns related to deviations from intended interventions, including variable adherence to the intervention recommendations.
Nayak et al., 2023 [20]	The study used proper computer-generated randomization, had balanced baseline characteristics, prespecified outcomes, and reported an intention-to-treat analysis; some concerns remained regarding post-randomization exclusions.
Jacobs et al., 2023 [21]	This randomized crossover trial had block randomization, high data completeness, prespecified registered outcomes, and primarily objective CGM-based endpoints. However, some concerns remained regarding insufficient reporting of allocation concealment.
Avari et al., 2020 [22]	The study was rated as having some concerns overall due to insufficient reporting of allocation concealment, substantial participant attrition, early trial termination caused by external factors (device discontinuation), and operational limitations affecting study conduct. Although the outcomes were objective (CGM-based), the amount of missing data and study disruptions raise concerns regarding the reliability of the results.

## Data Availability

No new data were created or analyzed in this study. Data sharing is not applicable to this article.
